# Astragalus Membranaceus Treatment Protects Raw264.7 Cells from Influenza Virus by Regulating G1 Phase and the TLR3-Mediated Signaling Pathway

**DOI:** 10.1155/2019/2971604

**Published:** 2019-12-31

**Authors:** Yuxi Liang, Qiuyan Zhang, Linjing Zhang, Rufeng Wang, Xiaoying Xu, Xiuhua Hu

**Affiliations:** ^1^School of Life Sciences, Beijing University of Chinese Medicine, Beijing, China; ^2^School of Traditional Chinese Medicine, Beijing University of Chinese Medicine, Beijing, China

## Abstract

Influenza is an acute respiratory infection disease caused by the influenza virus. At present, due to the high mutation rate of influenza virus, it is difficult for the existing antiviral drugs to play an effective antiviral effect continually, so it is urgent to develop a new anti-influenza drug. Recently, more and more studies have been conducted on the antiviral activity of *Astragalus membranaceus*, but the specific antiviral mechanism of this traditional Chinese medicine is not clear. In this study, the results proved that the *Astragalus membranaceus* injection showed obvious anti-influenza virus activity. It could improve the survival rate of Raw264.7 cells which were infected with influenza virus, while it improved the blocking effect of influenza virus on cell cycle after infection, increased the SOD activity, and reduced the MDA content. At the same time, the innate immunity was affected by regulating the expression of TLR3, TAK1, TBK1, IRF3, and IFN-*β* in the TLR3-mediated signaling pathway, thus exerting its antiviral effect in vitro.

## 1. Introduction

Influenza is a seasonal respiratory tract infectious disease caused by influenza viruses, and its clinical manifestations include acute respiratory symptoms such as high fever, fatigue, and cough. Influenza can cause many complications; common pulmonary complications include bronchitis, viral pneumonia, secondary bacterial pneumonia, and acute respiratory distress syndrome (ARDS) [1]. Common extrapulmonary complications of influenza include viral myocarditis, ischemic heart disease, stroke, viral encephalitis, influenza-associated conjunctivitis, and acute kidney injury [2]. That is why influenza has such high morbidity and mortality.

Influenza virus is the main pathogenic pathogen of influenza. It is a negative sense, single-stranded RNA virus (‐ss RNA virus) and a member of the Orthomyxoviridae family, which can be divided into four types: A, B, C, and D according to different nuclear proteins [3, 4]. The structure of influenza virus can be divided into three parts: core, matrix protein, and envelope from the inside out. The inner core is composed of nuclear protein (NP) and single-stranded RNA (ssRNA), while the viral envelope contains two viral transmembrane glycoproteins: hemagglutinin (HA) and neuraminidase (NA) [5, 6]. HA plays an important role in viral invasion of host cells. The influenza virus life cycle is initiated by the recognition of sialic acid (SA) of the host cell glycoprotein by HA. The primary role of NA is to hydrolyze SA from virus and cellular glycoproteins, while the budding newly formed virions can be released from infected cells [7–10].

After the infection of influenza virus, the innate immunity plays a critical role in efficient and rapid limitation of viral infections as well as for adaptive immunity initiation. There are different pathogen-associated molecular patterns (PAMP) to recognize the influenza virus, including toll-like receptors (TLRs) which make a difference in this process [11, 12]. TLRs have emerged as key sensors of innate immunity, in that they can respond to multiple pathogenic microorganisms and activate the innate immunity system by recognizing different signaling pathways [13, 14]. TLR3, as an important member of the TLR family, has been demonstrated to serve as an essential pattern recognition receptor (PRR) that can detect and fend off some invading viral pathogens [15]. DsRNA is the molecular characteristic of most viruses in the process of the virus proliferation, and it can be produced as an intermediate product of virus replication. TLR3 can activate the TRIF-dependent pathway after the recognition of the dsRNA and induce the downstream signal protein TBK1 to be phosphorylated. The phosphorylated TBK1 further activates IRF3 and induces the phosphorylated IRF3 to translocate into nuclei, and then it induced the secretion of cytokines IFN-*β* against viral infection. During this process, some other antiviral kinases such as TAK1 are also involved in [16, 17].

*Astragalus membranaceus* (AM) is traditional Chinese medicine, which is the dry root of astragalus mongolicus or membranous astragalus. Saponins, flavonoids, and polysaccharides are believed to be the principle active constituents of AM [18, 19]. More studies had confirmed that AM has many functions, including regulating immune function [20–22], antiviral, anti-inflammatory, antioxidant [23–26], antitumor [27–31], and cardiovascular protection [32, 33]. The antiviral activity of AM is the focus of this study. In clinical practice, AM could be used to replace some western medicine for antiviral treatment, so as to reduce the toxic and side effects of western medicine treatment on human body. Therefore, further study on the mechanism of AM antiviral treatment can provide scientific basis for future drug targeting research and clinical medication.

## 2. Materials and Methods

### 2.1. Drug

The *Astragalus membranaceus* injection (AMI) was purchased from HeiLongJiang ZBD Pharmaceutical Co., Ltd. (Heilongjiang, China). The dosage form of AMI is injection, and the strength is 2 g/ml.

### 2.2. Cell Line and Cell Culture

The mouse macrophages Raw264.7 and the Madin–Darby canine kidney (MDCK) cells were obtained from the Cell Resource Center, Peking Union Medical College (Beijing, China). The cells were cultivated in the 25 cm^2^ cell culture flasks in DMEM (SH30022.01, Hyclone, Logan, Utah, USA) supplemented with 10% (v/v) FBS (11011-8611, Tianhang Biotechnology, Zhejiang, China) at 37°C in a humidified 5% CO_2_ atmosphere, and they were split 1 : 3 to 1 : 6 when the confluent was reached 80%∼90%.

### 2.3. Virus Amplification

The mouse influenza A virus strain A/FM/1/47 (H1N1) was provided by the Institute of Virology, Chinese Academy of Preventive Medicine. MDCK cells were cultivated in the 25 cm^2^ cell culture flasks, when the cells grew into a single layer with a density of 90%, 2 ml influenza virus were inoculated into the cell culture flask after being washed with sterile saline. Two hours later, 7 ml DMEM supplemented with 2% FBS was added to the flaks for the cell culture. The morphological changes of the cells were observed every day. When obvious cytopathic changes were observed, cells and supernatant were collected and freezing-thawed three times to obtain the standard strain of mouse H1N1 influenza virus and then stored in the refrigerator at −80°C for later use after determining titer.

### 2.4. Evaluation of Virus Infectivity Titer

The mouse influenza A virus strain A/FM/1/47 (H1N1) infectivity titer in MDCK cells was measured before further studies. According to Reed & Muench formula, we concluded that TCID_50_ of influenza virus was 10^−3.62^/0.1 ml. The dilution of virus that could cause lesions in half of the cells was 10^−3.62^. In this experiment, the virus solution was used when it was diluted 1000 times.

### 2.5. Cytotoxicity Assay

We tested the cytotoxicity of AM in Raw264.7 cells by the MTT assay (M8180, Solarbio, Beijing, China). Raw264.7 cells were seeded in a 96-well plate with 5000 cells/well at the logarithmic growth phase. Cells were divided into a normal control group treated with fresh culture medium alone and AMI groups treated with fresh culture medium with different concentrations of AMI at 37°C in a humidified 5% CO_2_ atmosphere for 24 h, 48 h, and 72 h, respectively. Then, MTT solution with the concentration of 0.5 mg/mL was added to each well and incubated at 37°C for 4 h. The MTT-formazan product was dissolved in DMSO (D2650, Sigma, St. Louis, Missouri, USA), and the OD values was estimated by measuring absorbance at 490 nm in an absorbance microplate reader (SpectraMax i3x, Molecular Devices, Silicon Valley, USA). The inhibitory rate was calculated by the formula: inhibitory rate (%) = (1−the average OD value of the treatment group/the average OD value of control group) × 100%. According to the results, IC_0_, IC_10,_ and IC_25_ of each time period were calculated which represented the drug concentration when the cell inhibitory rate was 0%, 10%, and 25%, respectively.

### 2.6. Anti-Influenza Virus Activities of AMI

Raw264.7 cells were seeded in a 96-well plate with 5000 cells/well at the logarithmic growth phase and cultured at 37°C in a humidified 5% CO_2_ atmosphere for 24 h. Cells were divided into four groups and treated with the following conditions: normal control group (saline + culture medium), AMI group (saline + culture medium supplemented with 9.5, 40, and 92 mg/ml AMI), virus control group (influenza virus + culture medium), and treatment group (influenza virus + culture medium supplemented with 9.5, 40, and 92 mg/ml AMI). Briefly, after treatment, cells were incubated for another 72 hours. Then, the supernatant of each group was removed for later use. Finally, the cell viability was assessed by the MTT assay. The inhibitory rate was calculated by the formula and analyzed using the method of statistics.

### 2.7. Flow Cytometry Assay

The proliferation cycle of Raw264.7 cells was detected by flow cytometry. The cells were inoculated in 6-well plates using the same procedure as mentioned above. After 72 h of incubation, the cells were harvested with 0.25% Trypsin–EDTA (SH30042.01, Hyclone, Logan, Utah, USA) and centrifuged at 1000 r/min for 5 min, then washed with PBS (P1010-2, Solarbio, Beijing, China) once, and fixed with 75% pre-cooled alcohol. The fixed cells were washed with PBS and centrifuged at 1000 r/min for 5 min, and then the supernatant was discarded. Collected cells were inoculated with 50 *µ*g/ml RNA enzyme (RN25-1, Magen, Guangzhou, China), and then 25 *µ*g/ml PI (KGA214, KeyGEN BioTECH, Jiangsu, China) was added to incubate cells at room temperature in a dark place for 15 min, followed by detection using a flow cytometer.

### 2.8. Superoxide Dismutase (SOD) Assay and Malondialdehyde (MDA) Assay

Under the guidance of the instructions in SOD assay (A001-3, Nanjing Jiancheng Bioengineering Institute, Jiangsu, China) and MDA assay (A003-1, Nanjing Jiancheng Bioengineering Institute, Jiangsu, China), the working liquid was prepared and added to each group of the preserved cell supernatant. After the reaction, the absorbance value was detected with a microplate reader. The content of SOD and MDA was calculated by the formula in the instructions.

### 2.9. Western Blot Assay

The cells were inoculated in cell culture flasks using the same procedure as mentioned above. After 72 h of incubation, the cells were washed by 4°C precooled PBS twice and lysed with the RIPA lysis buffer (R0020, Solarbio, Beijing, China) supplemented with protease inhibitor cocktail for 30 min. The total proteins were gained by centrifugation at 12000 g for 5 min and quantified with the Bicinchoninic Acid (BCA) Protein Assay Kit (PC0020, Solarbio, Beijing, China). Equal amounts of protein were subject to electrophoresis on 8% SDS-polyacrylamide (SDS-PAGE) gels and transferred to a polyvinylidene fluoride (PVDF) membrane. The indicated primary antibodies were incubated overnight at 4°C, including TLR3 (Anti-TLR3, ab62566, Abcam Inc, Cambridge, UK), TAK1 (Anti-TAK1, bs-3585R, Bioss, Beijing, China), TBK1 (Anti-NAK/TBK1, ab40676, Abcam Inc, Cambridge, UK), IRF3 (IRF-3 Antibody, sc-33641, Santa Cruze, Dallas, Texas, USA), NS1 (NS1-23-1, sc-130568, Santa Cruze, Dallas, Texas, USA), IFN-*β* (Anti-IFN beta, bs-0784R, Bioss, Beijing, China), and *β*-actin (Beta Actin Antibody, 66009-1-Ig, Proteintech Group, Chicago, USA). Then, they were washed by tris buffered saline Tween (TBST) and incubated with the corresponding secondary antibodies at 37°C for 1 h. Finally, ECL solution (Solarbio, Beijing, China) was added and the protein was left in the dark room for exposure, development, and fixation.

### 2.10. Statistical Analysis

The professional statistical software Statistical Product and Service Solutions (SPSS) 20 was used for data analysis in this experiment. All data were presented as mean ± standard deviation (SD) and statistically analyzed by one-way analysis of variance (ANOVA). *P* < 0.05 was considered to indicate a statistically significant difference. All experiments were repeated three times.

## 3. Results

### 3.1. Drug Cytotoxicity of Different Concentrations of AMI in Raw264.7 Cells

**Figure 1:**
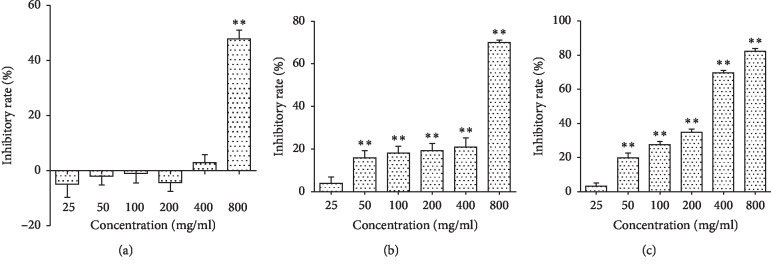
Effect of AMI on the cell proliferation of Raw264.7. Raw264.7 cells were treated with different concentrations (25, 50, 100, 200, 400, and 800 mg/ml) of AMI for 24 h (a), 48 h (b), and 72 h (c). ^*∗∗*^*P* < 0.01, compared with the normal control group.

The cells were treated with different concentrations of Astragalus membranaceus injection (AMI) for 24 h, 48 h, and 72 h. The results indicated that AMI exerted the cytotoxicity in Raw264.7 cells, and it can reduce the cell viability in a concentration-dependent and time-dependent manner. As shown in Figure 1, after 24 h of treating with AMI, cell proliferation was inhibited when the concentration was above 400 mg/ml. After the action of AMI on cells for 48 h and 72 h, it showed cytotoxic effects when the concentration was above 25 mg/ml. The drug cytotoxic effect of AMI in Raw264.7 cells increased with the time and concentration. According to the statistical software, the corresponding IC_0_, IC_10,_ and IC_25_ of each time period were calculated, as shown in Table 1. The IC_0_, IC_10,_ and IC_25_ of the AMI involved in this experiment were 9.5 mg/ml, 40 mg/ml, and 92 mg/ml, respectively.

### 3.2. Anti-Influenza Virus Effects of AMI In Vitro

**Figure 2:**
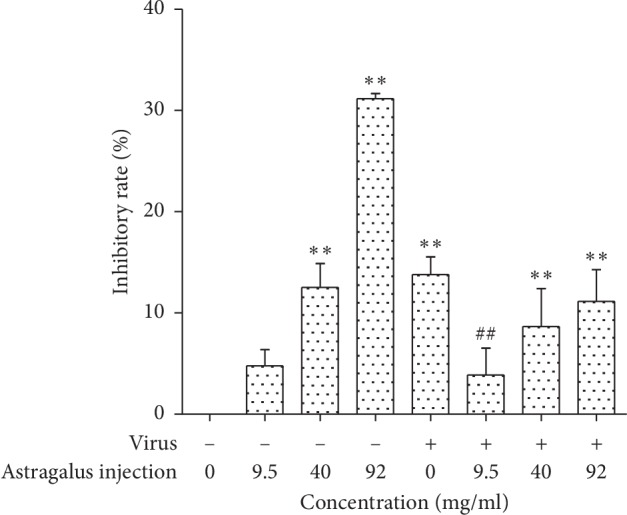
Results of anti-influenza Virus activities of AMI for 72 h. ^*∗∗*^*P* < 0.01, compared with the normal control group. ^##^*P* < 0.01, the treatment group compared with the virus control group.

We investigated the anti-influenza virus effects of AMI. As shown in Figure 2, in the virus control group, cell proliferation was significantly inhibited and with an inhibition rate of 13.8%. The inhibition effect of virus on cell proliferation was significantly reduced when the cells were treated with different concentrations (9.5, 40, and 92 mg/ml) of AMI for 72 h. The proliferation inhibitory rate of the virus infected group treated by 9.5 mg/ml AMI had statistical difference compared with the virus control group. These three different concentrations of AMI showed the antiviral effect at certain degrees.

### 3.3. AMI Can Improve the Cell Cycle of Raw264.7 Cells Infected with Influenza Virus In Vitro

**Figure 3:**
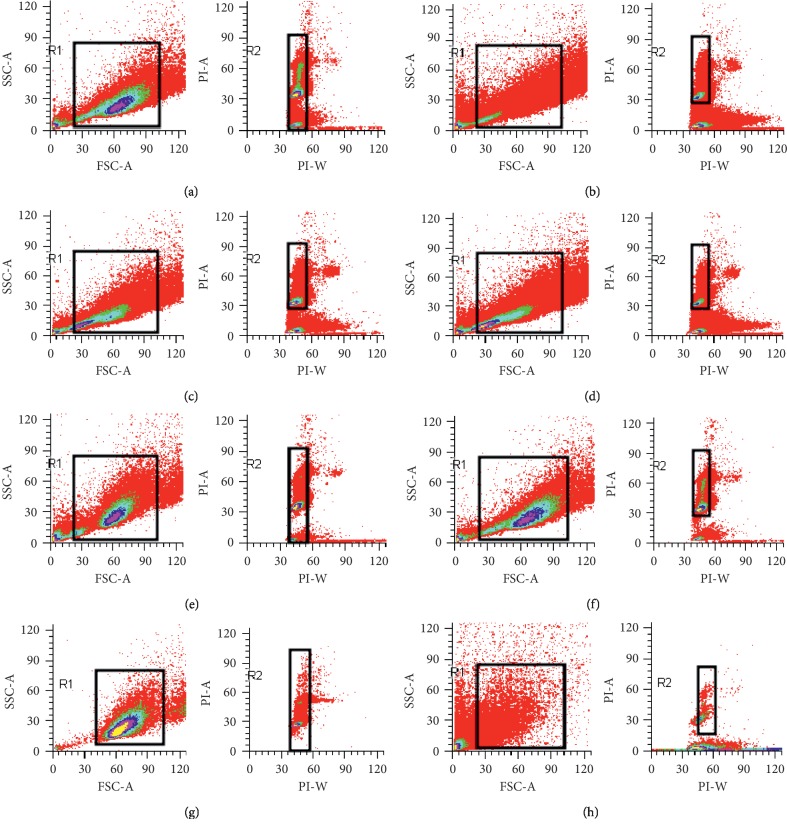
Effect of AMI on the cell cycle of Raw264.7 cells infected with influenza virus. (a) Normal control group. (b) AMI group (9.5 mg/ml). (c) Virus + AMI (40 mg/ml). (d) Virus + AMI (92 mg/ml). (e) Virus control group. (f) Virus + AMI (9.5 mg/ml). (g) AMI group (40 mg/ml). (h) AMI group (92 mg/ml).

To clarify the antiviral mechanism of AMI, we analyzed the data of cell cycle by flow cytometry. As shown in Table 2 and Figure 3, compared with the percentage of G1 phase cells in the normal control group (65.74%), the percentage of G1 phase cells in the treatment group had no statistical significance (66.60%, 67.58%, and 66.68%), but there was significant statistical difference when they were compared with the percentage of G1 phase cells in the virus control group (73.14%). The result indicated that AMI could improve or eliminate G1 arrest caused by the virus and restored the cell cycle to the level of the normal control group.

### 3.4. Effects of AMI on the Content of SOD and MDA in Raw264.7 Cells Infected with Influenza Virus

**Figure 4:**
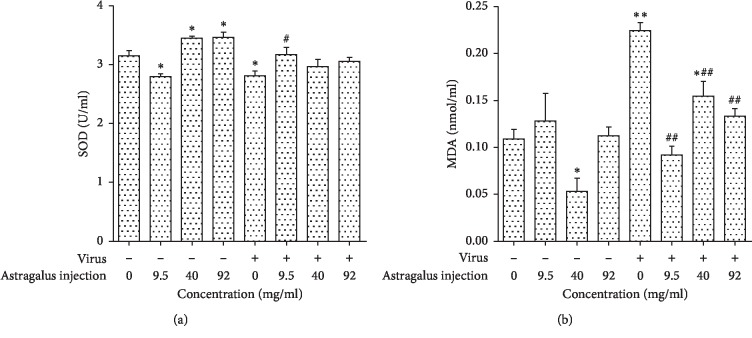
Effects of AMI on the content of SOD (a) and MDA (b) in Raw264.7 cells infected with influenza virus. ^*∗*^*P* < 0.05 and ^*∗∗*^*P* < 0.01 compared with the normal control group. ^#^*P* < 0.05, ^##^*P* < 0.01, the treatment group compared with the virus control group.

To explore the antiviral mechanism of AMI, we next investigated the influence of AMI on the content of SOD and MDA in Raw264.7 cells. As shown in Figure 4, the result revealed that the content of SOD was decreased significantly (*P* < 0.05), while the content of MDA was increased in cells after the infection by influenza virus (*P* < 0.01). In the absence of virus, a certain dose of AMI could induce the increase in SOD level in normal cells. Compared with the virus control group, the infected cells by influenza virus produced more SOD and less MDA after being treated with AMI (9.5 mg/ml, 40 mg/ml, and 92 mg/ml).

### 3.5. Effects of AMI on the Expression of Protein (TLR3, TAB1, TBK1, IRF3, and IFN-*β*) in Raw264.7 Cells Infected with Influenza Virus

**Figure 5:**
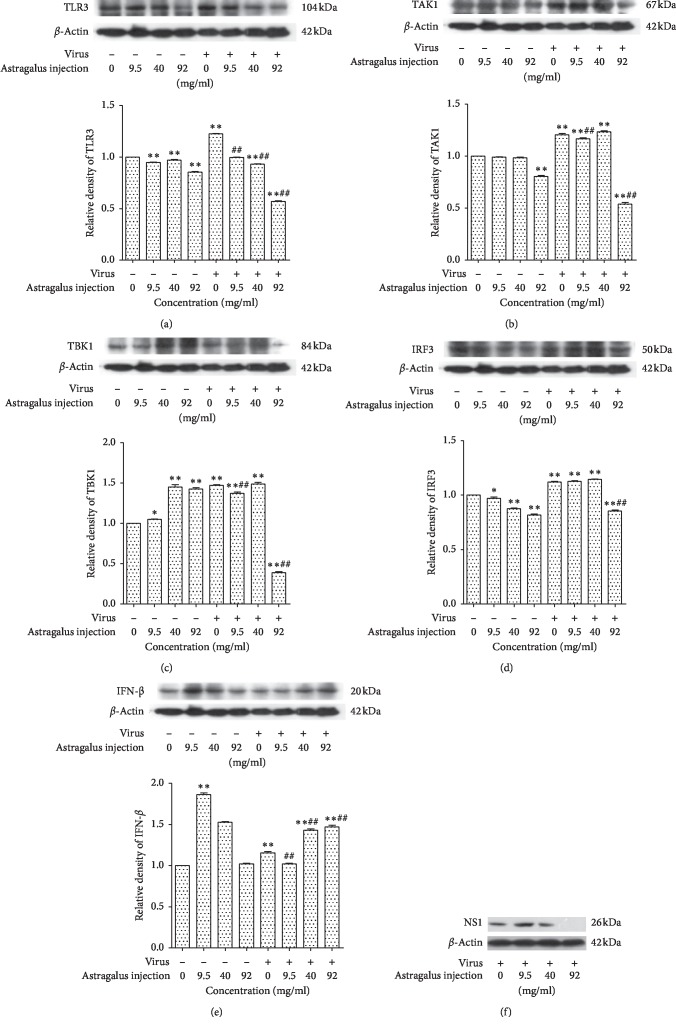
Effects of AMI on the protein expression of vital genes TLR3 (a), TAK1 (b), TBK1 (c), IRF3 (d), IFN-*β* (e), and NS1 (f) in Raw264.7 cells in response to influenza virus stimulation. ^*∗*^*P* < 0.05 and ^*∗∗*^*P* < 0.01 compared with the normal control group. ^##^*P* < 0.01, the treatment group compared with the virus control group.

The effects of AMI on the protein expression of TLR 3, TAK1, TBK 1, IRF 3, IFN-*β*, and NS1 are displayed in Figure 5. The change of NS1 protein expression indicated that the influenza virus had infected cells and the AMI could affect the expression of NS1. In the absence of influenza virus, the AMI (at a dose of 9.5 mg/ml or 40 mg/ml) could downregulate the expression of TLR3 and IRF3 and upregulate the expression of TBK1 and IFN-*β*, while the expression of TAK1 had no significant change; the AMI (at a dose of 92 mg/ml) could downregulate the expression of TLR3, TAK1, and IRF3 and upregulate the expression of TBK1, while the expression of IFN-*β* had no significant change. Compared with the normal control group, the expression levels of all proteins (TLR3, TAK1, TBK1, IRF3, and IFN-*β*) were increased after infection with influenza virus. In the presence of influenza virus, the expression of TLR3, TAK1, TBK1, and IFN-*β* could be downregulated by treatment with AMI at a dose of 9.5 mg/ml, while the expression of IRF3 had no significant change; the AMI at a dose of 40 mg/ml could downregulate and upregulate the expression of TLR3 and IFN-*β*, respectively, and had no significant influence on TAK1, TBK1, and IRF3; the AMI at a dose of 92 mg/ml could downregulate the expression of TLR3, TAK1, TBK1, and IRF3, while the expression of IFN-*β* was upregulated.

## 4. Discussion

As we all know, *Astragalus membranaceus* (AM) was originally described in the Sheng Nong's Herbal Classic which is the Classic of Herbal Medicine in ancient traditional Chinese medicine practice. It has a long history of medicinal use as one of the most important qi-tonifying herbs in traditional Chinese medicine [34]. Recently, AM is widely used in clinic treatment. Pharmacological research indicates that there are some important chemical substances in AM which is valued for its various effects. This fact is being increasingly substantiated by pharmacological studies showing that AM can increase the body's metabolism and immune function, increase hypoglycemia, and reduce hyperglycemia. Moreover, it also has some other significant effects including antioxidant, anti-inflammatory, anticancer, expectorant, diuretic, and antiviral effect [35]. The above fully indicates that AM has significant medicinal value. Our study demonstrated that AM showed obvious anti-influenza virus activity, and they could improve the survival rate of Raw264.7 cells which were infected with influenza virus.

Cell cycle refers to the whole process of cell life during which a cell undergoes duplication and division leading to the generation of two daughter cells, and it is the basic process of cell life activities. The eukaryotic cell cycle is generally divided into four stages: gap 1 phase (G1), synthesis phase (S), gap 2 phase (G2), and mitotic phase (M) [36]. There are key regulatory points in different phases of the cell cycle, the most important of which are the regulatory points in the G1 phase and S phase. Some studies have shown that viruses can disrupt the cell cycle after infecting cells and provide the best environment for self-replication by changing the regulatory points of cell cycle. As the microorganism living in cells, viruses lack protease needed for self-replication and proliferation, so they need to rely on proteins in host cells for replication. These proteins gather in large quantities in the S phase of cell cycle, indicating that the infection of cells by virus can prevent the transformation of cells into the S phase and leave them stuck in the G1/S transition period. Flow cytometry (FCM) is the most commonly used method for detecting cell cycle and has extensive application prospects in many fields such as basic medicine, clinical medicine, and scientific research. Therefore, in our study, flow cytometry was used to detect the effect of the AMI on the cell cycle of Raw264.7 which was infected with influenza virus. Flow cytometry confirmed that the infection of Raw264.7 by influenza virus could inhibit the transformation of cells into the S phase and stuck them in the G1 phase. After treating with different concentrations of AMI, the situation about the G1 phase block caused by influenza virus could be improved significantly.

Superoxide dismutase (SOD) is an important antioxidant metalloenzyme in an organism, which can repair the damaged cells and recover the damage caused by free radicals to human. SODs form the front line of defense against reactive oxygen species (ROS)-mediated injury [37]. It has been proved that SOD can delay the time of death and improve the survival rate of mice which were infected with influenza virus [38]. Malondialdehyde (MDA) is an end product of the radical-initiated oxidative decomposition of polyunsaturated fatty acids, and its content can reflect the degree of lipid peroxidation in the organism. Therefore, it is frequently used as a biomarker of oxidative stress [39]. In our study, the data showed that the infection of Raw264.7 by influenza virus could lead to the decrease of SOD and the increase of MDA. After the treatment of a certain dose of AMI, the SOD activity increased in infected cells and was restored to a normal level, and at the same time, the content of MDA was reduced. Thus, AMI protect cells from injury by influenza virus.

**Figure 6:**
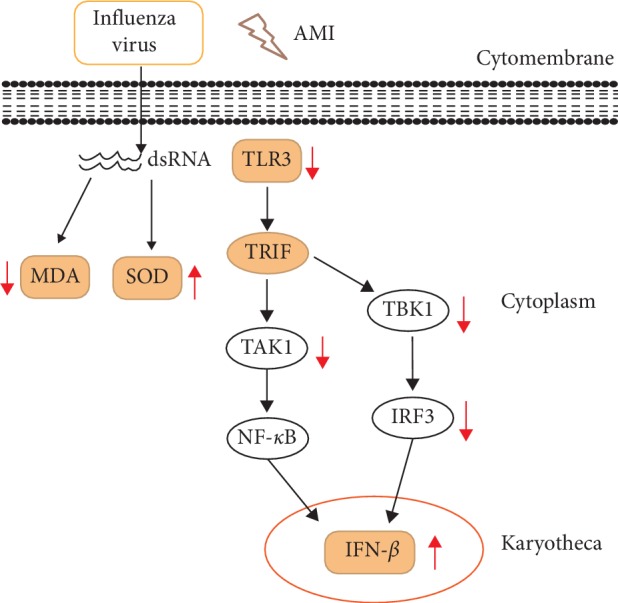
Effects of AMI (92 mg/ml) on the mechanism of Raw264.7 cells infected with influenza virus.

The TLR antiviral signaling pathways are classified into MyD88-dependent and non-MyD88-dependent pathways. TLR3 uses a TRIF-dependent pathway which can bind to various downstream signaling molecules (TBK1, TAK1, IKK*α*, etc.), and in this way, the IRF3 and NF-*κ*B signaling pathways are activated to promote the innate immunity against viruses. In the infected cells, TLR3 can recognize dsRNA and activate TBK1 by acting on the TRIF-dependent pathway; then, phosphorylated IRF3 is induced to translocate into nuclei; finally, it can induce the secretion of cytokines IFN-*β* against viral infection [40]. In our study, the data of western blot proved that infection of influenza virus could increase the expression of the innate immune system-related factors TLR3, TAK1, TBK1, IRF3, and IFN-*β* in Raw264.7 cells. Furthermore, we preliminarily confirmed that AMI could play an antiviral role by regulating the TLR3-TBK1-IRF3 signaling pathway. During this progress, the expression of TLR3 and TLR3-related downstream signaling factors were reduced. In addition, in the absence of influenza virus, the result showed that AMI could affect the expression of TAK1 which was located at the upstream of the NF-*κ*B signaling pathway. NF-*κ*B can be activated by TAK1 and then induce the expression of IFN-*β*, thus exerting immune effects. Our studies suggested that AMI might also exert antiviral effect by affecting the signal pathway of TLR3-TAK1-NF-*κ*B, and we will conduct further research and verification in follow-up experiments. In addition to the abovementioned results, the effects of different concentrations of AMI (9.5 mg/ml, 40 mg/ml, and 92 mg/ml) on infected cells were not consistent. With the increase of AMI dose, IFN-*β* expressed in certain degrees, and the AMI at a dose of 92 mg/ml could increase the expression of IFN-*β*, as is shown in Figure 6. These results suggested that the antiviral effect of AMI might be a multitarget and bidirectional mechanism, which was affected by drug concentration.

In summary, the present study explored the antiviral effects and the mechanism of AMI in mouse Raw264.7 cells. Experimental results clarified that AMI could exert its antiviral effect by influencing the cell proliferation cycle, the content of SOD and MDA, and the expression of related factors in the TLR3-mediated signaling pathway. The antiviral mechanism of AMI was clarified at the cellular and molecular levels, and it provides the experimental evidence for clinical guidance of drug use. Finally, based on the abovementioned experimental results, we hypothesized that AMI could affect the cell proliferation cycle and the levels of SOD and MDA in cells after infection of influenza virus strains in mice, as well as the expression of the TLR3 signaling pathway-related factors, so the hypothesis of whether there is a necessary connection between them will be tested further.

## Figures and Tables

**Table 1:** IC0, IC10, and IC25 of AMI in each time period.

Drug action time	IC0 (mg/ml)	IC10 (mg/ml)	IC25 (mg/ml)
24 h	301	412	578
48 h	190	338	472
72 h	9.5	40	92

**Table 2:** Effects of AMI on the cell cycle of Raw264.7 cells ($\bar{x}$ ± *s*·*n* = 3).

Group	Concentration (mg/ml)	Inhibitory rate (%)	The percentage of cells in each phase (%)		
			G1 phase	S phase	G2 phase
Normal control group	0		65.74 ± 2.32	30.89 ± 2.49	3.37 ± 0.17
Virus control group	0		73.14 ± 1.96^*∗∗*^	22.53 ± 1.91^*∗∗*^	4.33 ± 0.06
AMI group	9.5	0	76.94 ± 1.02^*∗∗*^	20.19 ± 0.17^*∗∗*^	2.87 ± 1.19
	40	10	77.20 ± 0.09^*∗∗*^	18.81 ± 0.39^*∗∗*^	3.99 ± 0.48
	92	25	83.87 ± 0.51^*∗∗*^	13.32 ± 0.51^*∗∗*^	2.82 ± 0.01
Treatment group	9.5	0	66.60 ± 1.43^##^	29.97 ± 1.41^##^	3.43 ± 0.02
	40	10	67.58 ± 0.16^##^	28.23 ± 0.10^##^	4.18 ± 0.06
	92	25	66.68 ± 1.74^##^	28.92 ± 2.97^##^	4.39 ± 1.24

Note. ^*∗∗*^*P* < 0.01, compared with the normal control group. ^##^*P* < 0.01, the treatment group compared with the virus control group.

## Data Availability

The data used to support the findings of this study are available from the corresponding author upon request.
